# Associations of glycaemia‐related risk factors with dementia and cognitive decline in individuals with type 2 diabetes: A systematic review and meta‐analysis

**DOI:** 10.1111/dme.70123

**Published:** 2025-08-19

**Authors:** Mahtab Tabesh, Julian W. Sacre, Kanika Mehta, Lei Chen, Seyedeh Forough Sajjadi, Dianna J. Magliano, Jonathan E. Shaw

**Affiliations:** ^1^ Baker Heart and Diabetes Institute Melbourne Victoria Australia; ^2^ Baker Department of Cardiovascular Research, Translation and Implementation La Trobe University Melbourne Victoria Australia; ^3^ School of Public Health and Preventive Medicine Monash University Melbourne Victoria Australia

**Keywords:** Alzheimer's disease, dementia, diabetes complications, glycaemic control, hypoglycaemia, meta‐analysis, type 2 diabetes

## Abstract

**Aims:**

To quantify prospective associations of glycaemia‐related factors with cognitive decline and all‐cause dementia and its subtypes in people with type 2 diabetes.

**Methods:**

We systematically searched Embase and MEDLINE (January 2000–October 2024) for studies in people with diabetes reporting longitudinal associations of a relevant exposure (i.e. hypoglycaemia, HbA1c, HbA1c variability or diabetes duration) with any of these outcomes: cognitive decline, all‐cause dementia, Alzheimer's disease (AD) or vascular dementia (VaD). Data were meta‐analysed using a random‐effects model followed by meta‐regression if appropriate.

**Results:**

Forty studies representing 7,076,724 individuals with diabetes were included. Hypoglycaemia was significantly associated with 49% and 31% higher risks of all‐cause dementia and AD, respectively. The pooled effect size did not significantly vary according to age, sex, diabetes duration, smoking, follow‐up length, comorbid hypertension, kidney disease, dyslipidaemia or stroke (all *p* > 0.05). A positive association existed between hypoglycaemia frequency and all‐cause dementia, with maximum hazard ratios (HRs) of 2.36–2.60 in the highest exposure group. HbA1c showed a positive risk gradient for all‐cause dementia, with maximum significant HRs of 1.40–3.88 for the highest category, while only three studies were available for meta‐analysis, with a pooled HR (95% CI) of 1.18 (0.97, 1.45). HbA1c variability and diabetes duration were each significantly associated with a higher risk of dementia. Limited evidence supported a relationship between glycaemia‐related factors and cognitive decline.

**Conclusions:**

Having a history of hypoglycaemia, longer diabetes duration, and higher HbA1c levels and variability were related to higher dementia risk in people with type 2 diabetes.

**What's new?**

**What is known?**

- People with type 2 diabetes are at higher risk of dementia.
- Dementia is a significantly burdensome complication and among the leading causes of death in this population.

**What did we find?**

- Glycaemia‐related factors, that is hypoglycaemia, high HbA1c, HbA1c variability and long duration of diabetes are all associated with this higher risk.

**What are the implications?**

- Glycaemic control in individuals with type 2 diabetes who are susceptible to dementia should focus on avoiding hypoglycaemia, very high HbA1c levels and high fluctuations in HbA1c.
- Longer diabetes duration is associated with higher dementia risk; therefore, people with longstanding diabetes require good management of modifiable risk factors.

## 1 INTRODUCTION

As populations age, especially in high‐income countries, the number of individuals living with cognitive decline and dementia continues to rise.^1^ Diabetes is a widely recognised risk factor for cognitive disorders, and individuals with diabetes are reported to have an approximately 1.6 times higher risk of dementia compared to people without diabetes.^2^ In recent years, cognitive dysfunction has gained significant attention as an emerging complication of diabetes; indeed, dementia has become one of the leading causes of death in type 2 diabetes.^3^

Nevertheless, it remains unclear which diabetes‐specific factors may be responsible for the excess dementia risk observed among people with diabetes. Glycaemic control (indicated by HbA1c), glycaemic variability, hypoglycaemia and diabetes duration are all implicated in the development of traditional diabetes complications, but their relative importance to cognitive impairment and dementia is not well established.^4^, ^5^, ^6^ Resolving this issue is a high priority given both the lack of specific therapeutic targets for diabetes‐related cognitive impairment and the unique diabetes care considerations applicable to the older adults most affected; that is less stringent glycaemic control and minimisation of polypharmacy compared with younger individuals with type 2 diabetes.^7^

In this context, we aimed to conduct a comprehensive systematic review and meta‐analysis of the literature to evaluate the associations of glycaemia‐related factors, that is hypoglycaemia, HbA1c levels, HbA1c variability and diabetes duration, with the risk of all‐cause dementia and its subtypes or decline in cognitive function in people with type 2 diabetes. We further explored the potential moderators of these associations via meta‐regression.

## 2 METHODS

### 2.1 Protocol

Our protocol was registered on the Prospective Register of Systematic Reviews (PROSPERO) with registration number CRD42023462487.^8^ This systematic review focused solely on the association of glycaemia‐related factors and cognitive outcomes outlined in the protocol. Reporting was conducted according to the Preferred Reporting Items for Systematic Reviews and Meta‐Analysis (PRISMA).^9^

### 2.2 Search strategy

We searched MEDLINE and Embase for research articles published from January 2000 to October 2024 (Table S1 shows the detailed search strategy). We complemented this search by reviewing the reference lists of relevant reviews to identify additional studies that may have been missed. We included cohort studies, studies based on registries and observational analyses of clinical trials among people with total diabetes or type 2 diabetes. We excluded studies on people with type 1 diabetes or gestational diabetes mellitus, non‐human or low sample size (<200 participants) studies, and those not published in English.

### 2.3 Exposures and outcomes

We investigated the associations of hypoglycaemia, HbA1c, HbA1c variability and diabetes duration with all‐cause dementia, along with its subtypes or decline in cognitive function. All studies reporting these associations (whether assessed as the primary exposures or as covariates) were included. We included studies that investigated hypoglycaemia, defined based on blood glucose, as well as studies focusing on severe hypoglycaemia, characterised by the need for hospitalisation or assistance from another person to administer carbohydrates. Various metrics of HbA1c variability, including standard deviation (SD), coefficient of variation (CV) or average real variability (ARV) were included. The outcomes of interest were decline in cognitive function based on various cognitive tests (including the domains of global function, executive function, processing speed and memory) and clinical diagnosis of all‐cause dementia or its main subtypes, including Alzheimer's disease (AD) and vascular dementia (VaD).

### 2.4 Study selection

Two independent reviewers screened the title/abstract of retrieved studies followed by a full‐text screening based on pre‐defined eligibility criteria. In both stages, in cases of disagreements, a third reviewer was consulted to reach a consensus.

### 2.5 Assessment of risk of bias

For each included study, risk of bias was assessed using a modified Newcastle‐Ottawa Assessment Scale (NOS) for cohort studies as outlined in a previous publication.^10^ A NOS score of 1–4, 5–6 and 7–9 was considered high, moderate and low risk of bias, respectively. Each study was scored twice by independent reviewers, and cases of conflict were resolved by consulting a third reviewer.

### 2.6 Data extraction, aggregation and curation

Study characteristics along with details on exposure and outcome measurements were extracted. Measures of association included beta estimates, odds ratios (OR) and hazard ratios (HR) from multiple regression analyses. Where multiple studies were found to have reported on the same source of data, the one covering a longer time period or a larger sample size was selected. When studies reported on two different methods of measuring a variable (i.e. categorical and continuous), both sets of data were retained. To improve uniformity of data, when possible, relative risk metrics were recalculated to align with a consistent unit of measurement for the exposure variables (e.g. one percentage point increase in HbA1c instead of one mmol/mol increase). In studies reporting results from more than one regression model, we selected the effect estimate from the simplest model that at least adjusted for age and sex. This approach aimed to enhance homogeneity of included data given the wide variation in covariate selection in more complex models.

### 2.7 Statistical analysis

Statistical analyses were performed in Stata 18 (StataCorp) using ‘meta’ and ‘metan’ modules. Meta‐analyses were performed when exposures were evaluated on a dichotomous or a continuous basis, and at least three sets of data were available to pool. Otherwise, study‐specific data were reported without summary statistics in a forest plot or a table. In instances where HRs were not reported, ORs or sub‐distribution HRs were used instead.

A random‐effects model was used for meta‐analysis while variance was assessed with an empirical Bayes estimator. Variability among studies was quantified with tau^2^ and *I* ^2^, with *I* ^2^ measuring the proportion of total variation due to heterogeneity and tau^2^ estimating the absolute variance across study effects. For analyses with at least ten studies and high heterogeneity, random‐effects univariable meta‐regression models were used to explore the potential sources of heterogeneity. Covariates were selected based on a theoretical relation with dementia, including age, proportion of male participants, duration of diabetes, study follow‐up length, smoking status, dyslipidaemia, comorbid hypertension or chronic kidney disease, and history of stroke. Bubble plots were used to visually assess the magnitude of association between these covariates and effect size, while a Galbraith plot was used to visualise the magnitude of heterogeneity and identify outliers.

Publication bias was evaluated using funnel plots and Egger's test when at least ten studies were included. To ensure our findings were not impacted by potential temporal trends, we sought a relationship between publication year and effect size by arranging the studies in chronological order in addition to performing a cumulative meta‐analysis. This approach was taken only for meta‐analyses that included studies published over a period exceeding 10 years. To assess potential bias from taking the minimally adjusted models, we conducted a sensitivity analysis using effect estimates from the fully adjusted models. To minimise potential bias from using different measures of association, we conducted sensitivity meta‐analyses limited to studies reporting HRs. Finally, to assess the influence of individual studies on the pooled effect size, a leave‐one‐out sensitivity analysis was conducted by recalculating the effect size omitting studies from the estimation one at a time.

## 3 RESULTS

A total of 40 journal articles were included, representing 7,076,724 people with total diabetes or type 2 diabetes. Figure S1 summarises the workflow of study identification, screening and selection. The most studied glycaemia‐related factor was HbA1c with a total of 22 articles^7^, ^11^, ^12^, ^13^, ^14^, ^15^, ^16^, ^17^, ^18^, ^19^, ^20^, ^21^, ^22^, ^23^, ^24^, ^25^, ^26^, ^27^, ^28^, ^29^, ^30^, ^31^ followed by hypoglycaemia with 16 articles.^19^, ^20^, ^21^, ^23^, ^24^, ^32^, ^33^, ^34^, ^35^, ^36^, ^37^, ^38^, ^39^, ^40^, ^41^, ^42^ Eight articles contributed data for diabetes duration,^16^, ^17^, ^23^, ^29^, ^43^, ^44^, ^45^, ^46^ and five articles addressed HbA1c variability.^5^, ^6^, ^21^, ^23^, ^47^ Characteristics of included studies and NOS scores for individual studies are summarised in Table S2. A summary of the findings from the included studies is presented in Table S3. Most studies were found to have a low risk of bias, except for one with moderate risk, and none were excluded based on this score. The studies used slightly different definitions of hypoglycaemia, which are summarised in Table S4. Outcomes included change in specific domains or overall cognitive function as reported in 12 studies, while 28 studies reported on all‐cause dementia and its subtypes.

### 3.1 The association of hypoglycaemia with cognition

**FIGURE 1:**
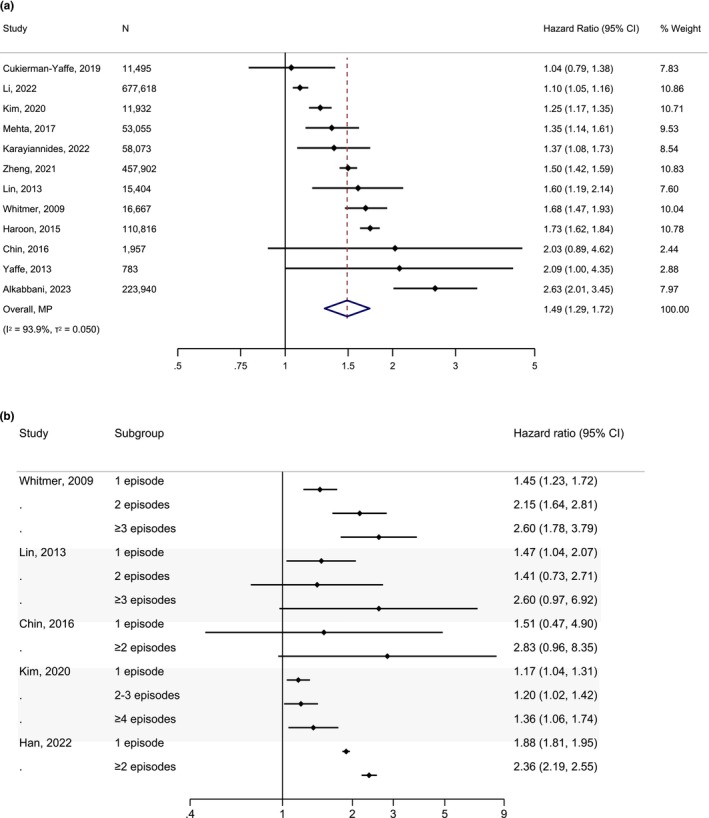
Association of hypoglycaemia with incident all‐cause dementia compared to no history of hypoglycaemia in people with type 2 diabetes. (a) Meta‐analysis of studies evaluating any history of hypoglycaemia. (b) Dose–response association of the number of hypoglycaemic events with incident all‐cause dementia.

**FIGURE 2:**
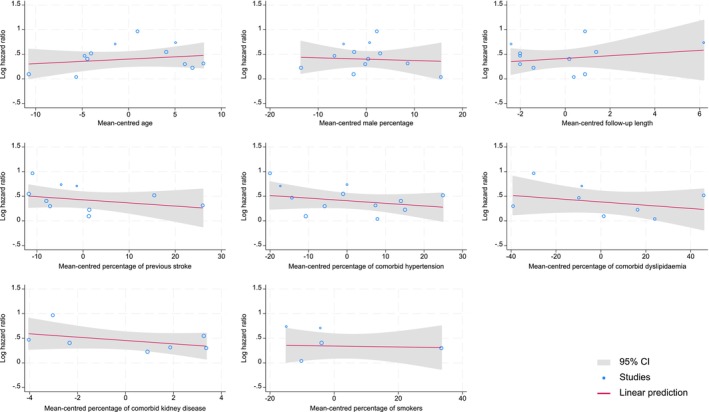
Meta‐regression plots evaluating the association of potential covariates with the effect size of studies.

Figure 1a illustrates the result from meta‐analysing the association of history of hypoglycaemia with all‐cause dementia. The pooled HR (95% CI) was 1.49 (1.29, 1.72) with a tau^2^ (95% CI) of 0.050 (0.019, 0.182) and an *I* ^2^ of 93.9%. Meta‐regression plots are presented in Figure 2, indicating that none of the explored covariates had a statistically significant association with the effect size in studies (all *p* > 0.05). The number of included studies for each plot ranged from five to all twelve studies depending on the availability of data. The Galbraith plot showed low heterogeneity with only one study falling outside the 95% CI region of the overall effect size (Figure S2). No publication bias was observed with the funnel plot (Figure S3) or Egger's test (*p* > 0.05). In a sensitivity analysis using effect sizes from fully adjusted models, the pooled HR (95% CI) remained very similar at 1.44 (1.23, 1.67) (Figure S4). No effect of calendar year was apparent with chronological ordering of studies or cumulative meta‐analysis (Figures S5 and S6). Leave‐one‐out sensitivity analysis revealed no disproportionate influence of any single study (Figure S7). In the sensitivity analysis limited to studies reporting HRs, a comparable pooled effect size of 1.57 (1.34, 1.84) was obtained (Figure S8).

Five studies evaluated the dose–response association of the number of hypoglycaemic episodes with the risk of all‐cause dementia. Trends toward increased risk with a progressively greater number of episodes were noted in all studies (Figure 1b).

When considering subtypes of dementia, a meta‐analysis of three studies focusing on AD showed a significant pooled HR (95% CI) of 1.31 (1.11, 1.54) with a tau^2^ (95% CI) of 0.069 (0.025, 0.237) and an *I* ^2^ of 16.3% (Figure S9). The single study examining hypoglycaemia and the risk of VaD reported a significant association (HR (95% CI) 1.29 (1.11, 1.49)).^37^ For dementia risk according to the number of hypoglycaemic episodes, a single study reported a HR (95% CI) of 1.83 (1.75, 1.92) with one episode and 2.41 (2.21, 2.64) with two or more episodes for AD and a HR (95% CI) of 1.91 (1.71, 2.13) with one episode and 1.97 (1.54, 2.52) with two or more episodes for VaD.^42^

Two studies assessed the association of hypoglycaemia with cognitive function. In one study, a global cognitive ability factor (*g*) was calculated based on several tests assessing memory, executive function, processing speed and nonverbal reasoning.^34^ Having a history of hypoglycaemia was not associated with having a lower *g* at four years follow‐up (OR (95% CI) 1.65 (0.99, 2.76) for the lowest tertile) or a steeper decline in *g* between baseline and follow‐up (OR (95% CI): 1.36 (0.82, 2.24)). In another study, the association of hypoglycaemia with the Montreal Cognitive Assessment (MoCA) score was studied. Those people reporting more than five episodes of hypoglycaemia were at significantly higher risk of developing mild cognitive impairment than were people reporting 5 or fewer episodes (OR (95% CI) 4.64 (1.28, 16.78)).^24^

### 3.2 The association of HbA1c level with cognition

**FIGURE 3:**
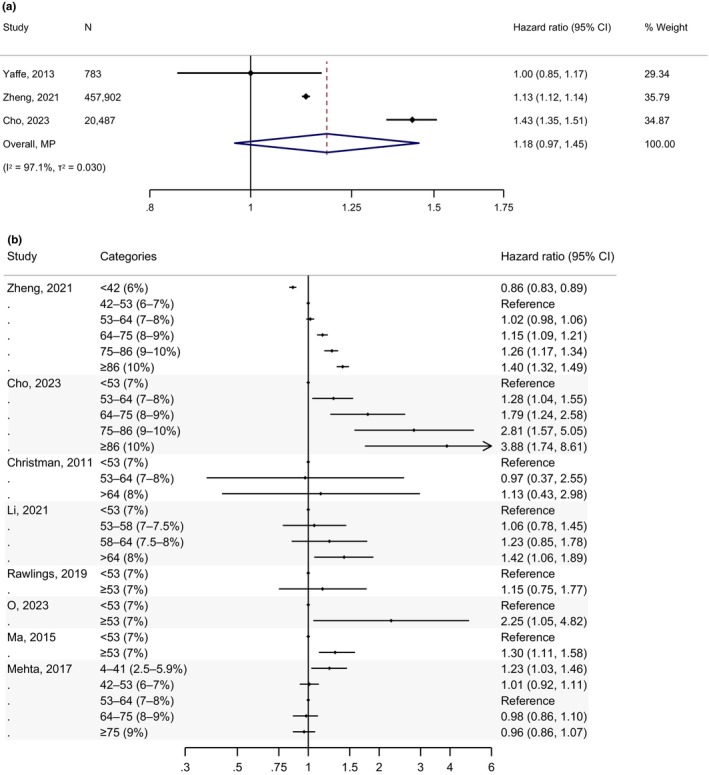
Association of HbA1c levels with incident dementia in people with type 2 diabetes. (a) Meta‐analysis of studies assessing HbA1c as a continuous variable. (b) Summary of studies assessing HbA1c as a categorical variable.

A meta‐analysis of three studies assessing all‐cause dementia risk per percentage point increase in HbA1c yielded a pooled HR of 1.18 (95% CI: 0.97, 1.45) (Figure 3a). Figure 3b shows that seven studies examined the association of HbA1c as a categorical variable with all‐cause dementia, most of which demonstrated a positive dementia risk gradient with HbA1c levels. Lowest risks were primarily associated with the lowest HbA1c ranges, while the greatest risks varied, with significant HRs ranging from 1.40 to 3.88 in the highest HbA1c categories relative to the reference range. One study found the highest risk within the 4–41 mmol/mol (2.5%–5.9%) HbA1c range, with other categories showing similar risks to the reference group (HbA1c 53–64 mmol/mol (7%–8%)).^19^ In the sensitivity analysis limited to studies reporting HRs, a pooled effect size of 1.09 (0.98, 1.22) was obtained (Figure S10). In the sensitivity analysis based on effect sizes from fully adjusted models, a pooled effect size of 1.16 (0.94, 1.44) was observed along with consistent associations between HbA1c categories and all‐cause dementia (Figure S11).

One study investigated the proportion of time that a person was in their individualised clinical guideline‐directed HbA1c target.^7^ This study reported a significant HR of 1.19 (1.16, 1.23) for the risk of all‐cause dementia in individuals with <20% in the target range compared to those with 80% or more in the target range. Additionally, 60% or greater time below the target range was associated with an increased risk of all‐cause dementia with a significant HR of 1.23 (1.19, 1.27), whereas 60% or greater time above the target range did not show a significant association.^7^

In a meta‐analysis of studies focusing on AD, a pooled HR of 1.11 (0.94, 1.30) was observed in the main analysis and a pooled HR of 1.02 (0.95, 1.10) in the sensitivity meta‐analysis limited to HRs (Figures S12a and S13). Two studies also evaluated categories of HbA1c, and both demonstrated risk gradients, with significantly increased risk of AD observed among those in the highest categories of HbA1c (Figure S12b). Only two studies evaluated the risk of VaD with HbA1c level. The results from these studies suggested a positive risk gradient with increasing HbA1c categories while one study reported an overall significant HR of 1.15 (1.12, 1.24) for each percentage point increase in HbA1c (Figure S14).^12^, ^16^

A total of 10 studies reported on the associations of HbA1c with various domains of cognition using a range of cognitive tests (Table S5).^11^, ^15^, ^18^, ^22^, ^24^, ^25^, ^26^, ^27^, ^28^, ^30^ Among them, four studies found a significant association of HbA1c with global cognition or executive function.^15^, ^26^, ^27^, ^28^ No significant associations were observed with measures of episodic memory or simple attention.

### 3.3 The association of HbA1c variability with cognition

Four studies evaluated the association of HbA1c variability with dementia or its subtypes and are summarised in Table 1.^6^, ^21^, ^47^, ^48^ Significant associations of HbA1c variability with the risk of all‐cause dementia were observed in all studies, irrespective of the metric studied (i.e. SD, CV, ARV). When considering AD, one study showed significant associations for HbA1c SD and CV in women only.^23^ Another study evaluated the association of HbA1c variability with AD by comparing tertiles of HbA1c CV and showed a significantly higher risk only for those with values in tertile 3 compared to tertile 1.^6^ Comparable results were observed when effect sizes from fully adjusted models were considered (Table S6).

**TABLE 1:** Association of HbA1c variability metrics with incident dementia and Alzheimer's disease in people with type 2 diabetes.

Outcome	Variability metric	Study	Subgroup	HR (95% CI)
All‐cause dementia	SD	Moran, 2024^47^		1.15 (1.12, 1.17)
	CV	Zheng, 2021^21^		1.02 (1.00, 1.04)^a^
		Moran, 2024^47^		2.56 (2.14, 3.06)
	ARV	Moran, 2024^47^		1.10 (1.09, 1.11)
AD	SD	Lee, 2021^48^	Women	1.14 (1.02, 1.27)
			Men	1.09 (0.94, 1.26)
	CV	Lee, 2021^48^	Women	1.01 (1.00, 1.02)
			Men	1.01 (0.99, 1.02)
		Li, 2017^6^	Tertile 1	Reference
			Tertile 2	1.08 (0.90, 1.28)^b^
			Tertile 3	1.60 (1.35, 1.89)^b^

*Note*: Results reported per unit change the variability metric unless specified otherwise.

Abbreviations: AD, Alzheimer's disease; ARV, average real variability; CI, confidence interval; CV, coefficient of variation; HR, hazard ratio; SD, standard deviation.

^a^ Results reported per SD change in CV.

^b^ Results reported compared to tertile 1.

One study evaluated the association of HbA1c variability with performance on cognitive tests.^5^ No statistically significant association was observed between HbA1c CV and cognitive tests of memory or executive function.

### 3.4 The association of duration of diabetes with cognition

**FIGURE 4:**
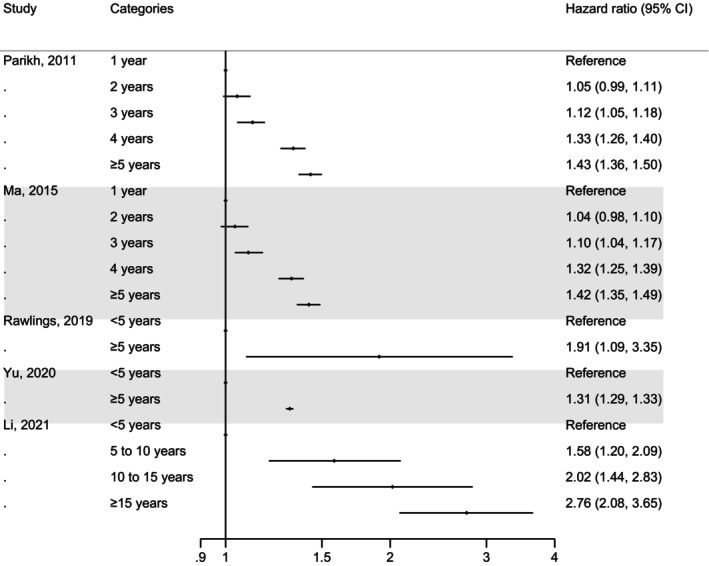
Association of diabetes duration and incident dementia in people with type 2 diabetes.

A total of eight studies evaluated the association of diabetes duration independent of age with the risk of all‐cause dementia or its subtypes.^16^, ^17^, ^23^, ^29^, ^43^, ^44^, ^45^, ^46^ Figure 4 illustrates results from five studies comparing categories of diabetes duration. A risk gradient was observed in studies comparing multiple categories of diabetes duration, with maximum significant HRs around 1.4 for a duration of five years or more compared with a one‐year duration,^17^, ^45^ and 2.76 for a duration of 15 years or more compared to five years.^16^ Similar results were observed when considering effect sizes from fully adjusted models (Figure S15). One study followed up people with newly diagnosed type 2 diabetes and observed a U‐shaped association between diabetes duration and dementia, with a decrease in risk for the first five years followed by an increase thereafter.^46^ Bruce et al. assessed the association of diabetes duration as a continuous variable with all‐cause dementia and showed a significant HR of 1.02 (1.00, 1.04) for each additional year of living with diabetes.^44^

Three studies examined the relationship between diabetes duration and subtypes of dementia (Figure S16). In one study, significant HRs of 1.30 (1.28, 1.32) and 1.32 (1.28, 1.37) were reported for AD and VaD, respectively, when comparing a diabetes duration of five years or more to <5 years.^43^ In another study, a risk gradient was observed with a maximum significant HR of 2.39 (1.44, 3.97) and 3.39 (2.12, 5.42) for AD and VaD, respectively, for having a diabetes duration of 15 years or more compared to <5 years.^16^ One study had a gender‐stratified analysis and showed a significant HR of 1.09 (1.03, 1.15) for women and 1.21 (1.11, 1.31) for men considering the risk of AD per each year increase in diabetes duration.^23^

## 4 DISCUSSION

In this study, we aimed to systematically evaluate the association of glycaemia‐related factors with the risk of dementia and its main subtypes, along with cognitive decline in people with type 2 diabetes. Our results support a positive association of having a history of hypoglycaemia, higher HbA1c levels, longer duration of diabetes and higher variability in HbA1c with the incidence of dementia and its subtypes of AD and VaD. However, studies assessing the association of HbA1c levels with the risk of decline in cognitive function are less consistent. This is the first meta‐analysis of longitudinal studies focusing on type 2 diabetes, including multiple glycaemia‐related risk factors and a wide range of cognitive outcomes, that is cognitive decline and all‐cause dementia, along with its subtypes.

Overall, our findings align with those of previous meta‐analyses.^49^, ^50^, ^51^ However, our review improved the robustness of the findings by addressing previous methodological limitations, such as combining results from studies on type 1 diabetes with those on type 2 diabetes, and pooling cross sectional and longitudinal data, which increases the risk of reverse causality. These limitations highlight the need for a more robustly designed systematic review, particularly given the substantial number of studies published in this field in recent years.

Based on this systematic review, hypoglycaemia demonstrated a consistent association with dementia, indicating a higher risk of developing dementia and its subtypes in individuals with a history of hypoglycaemic episodes. Furthermore, a dose–response relationship was observed, in which an increasing number of hypoglycaemic episodes corresponded to a higher risk of all‐cause dementia, AD and VaD. We explored potential sources of heterogeneity but observed no differential association of hypoglycaemia with all‐cause dementia in relation to common confounding factors such as age, sex, diabetes duration, smoking and presence of certain comorbidities such as hypertension, chronic kidney disease, dyslipidaemia and history of stroke in our meta‐regression. Moreover, there was no suggestion of significant levels of heterogeneity among included studies, as almost all reported individual effect sizes within the 95% CI of pooled effect size in meta‐analysis.

A linear association between the frequency of severe hypoglycaemic episodes and cognitive dysfunction was reported in a previous meta‐analysis suggesting a cumulative effect of neuronal damage with each episode.^51^ This damage is attributed to mechanisms such as selective neuronal cell death in the brain cortex and hippocampus, driven by oxidative stress, mitochondrial dysfunction, zinc release and excitotoxicity.^52^ The diabetic metabolic milieu can exacerbate this neuronal damage caused by hypoglycaemia. Impaired hippocampal synaptic function has been observed with recurrent hypoglycaemic events, further increasing the risk of cognitive dysfunction.^52^ Other possible explanations link hypoglycaemia to cognitive decline or dementia. Severe hypoglycaemia may serve as a marker of frailty and broader adverse outcomes without being causally related. Additionally, prodromal cognitive impairment could hinder diabetes self management, increasing the risk of hypoglycaemia.^53^

Regarding the association between HbA1c levels and dementia, almost all studies found a positive risk gradient with HbA1c level. One study reported a higher risk of dementia in the very low HbA1c category (4–41 mmol/mol (2.5%–5.9%)), while other HbA1c categories showed similar risks compared with the reference category (53–63 mmol/mol (7%–7.9%)). However, this analysis was based on HbA1c levels at the time of diabetes diagnosis rather than stabilised levels following treatment. Moreover, the study showed limited evidence of an association between some other common risk factors, such as hypertension, and dementia. Among the three studies evaluating HbA1c as a continuous variable, only one did not show an increased risk of dementia with higher HbA1c levels.^20^ This study, conducted in an older population (aged 70–79 years) with a 12‐year follow‐up, may have been influenced by the substantial competing risk of death, which reduced the analytic sample to only a quarter of the original cohort, potentially attenuating observed associations.

Studies examining cognitive decline through neurocognitive tests have identified a relationship between higher HbA1c levels and lower cognitive performance in domains such as global cognition and executive function, but not in attention or memory. However, great heterogeneity existed in the methods and range of neurocognitive tests even within each domain of cognition, making it difficult to compare results from different studies. Overall, it seems that the association of HbA1c with mild cognitive decline is not as robust as its association with dementia.

The mechanisms linking hyperglycaemia to cognitive impairment include increased oxidative stress, inflammation, the accumulation of advanced glycation end products, blood–brain barrier dysfunction, neuronal death and altered cerebral blood flow.^13^, ^14^, ^54^ Additionally, it has been suggested that mild cognitive impairment and dementia associated with type 2 diabetes may not be part of a single continuum but may involve distinct pathophysiological mechanisms, contributing to the observed inconsistencies.^55^ Finally, a non‐linear association between hyperglycaemia and cognitive dysfunction may not have been captured in studies relying solely on linear statistical models.^54^

HbA1c variability and the duration of diabetes both showed a positive association and a risk gradient with the incidence of all‐cause dementia, AD and VaD. HbA1c variability has been associated with other diabetes complications, with several mechanisms proposed to explain its role in increasing the risk of complications. Glycaemic variability has been associated with atherosclerosis of cerebral arteries, cerebral ischaemia and severe internal carotid artery siphon stenosis, all of which increase the risk of dementia.^50^ Additionally, fluctuations in HbA1c levels may indicate poor quality of care or inadequate medication adherence.^50^, ^56^ In individuals with longer diabetes duration, factors such as insulin resistance, chronic inflammation, oxidative stress and neurovascular dysfunction contribute to a higher incidence of dementia.^13^, ^57^

This is the first systematic review to consider all‐cause dementia along with its subtypes. Currently, relatively few studies have examined the association of risk factors with specific dementia subtypes in type 2 diabetes. A harmonisation of six community‐based autopsy studies has shown a high co‐occurrence of AD and VaD, referred to as mixed dementia, particularly in older populations.^58^ Notably, none of the reviewed studies in the current analysis used biopsy‐confirmed diagnoses, and the reliance on clinical records, neurocognitive testing or imaging techniques introduces uncertainty in accurately assigning dementia subtypes. Despite these limitations, the current evidence suggests that glycemia‐related factors are more strongly associated with VaD than with AD, aligning with their established links to other vascular diseases. However, there can be a potential bias toward diagnosing VaD in people with diabetes, particularly in studies based on clinical records.

A key strength of this study was its comprehensive scope, encompassing various aspects of cognition, from subclinical cognitive dysfunction to all‐cause dementia and its subtypes. The exclusive focus on longitudinal studies employing covariate‐adjusted statistical analyses further enhances the robustness and reliability of the evidence for the observed associations. Nevertheless, this systematic review also had some limitations. High methodological variability among the included studies made it difficult to compare results of different studies, especially those examining decline in cognitive function. It also hindered pooling results from studies that examined the outcomes based on inconsistent exposure categories. Another limitation was our inability to examine the differential role of risk factors in mid‐life onset dementia compared with later‐life onset. There is evidence that risk factors for dementia may differ by age of onset, which can possibly explain some of the heterogeneity in findings. Moreover, this review focused on people with type 2 diabetes, while a separate review in those with type 1 diabetes is warranted, as comorbidity profiles may vary between different diabetes types. Furthermore, the limited number of studies investigating dementia subtypes weakened the strength of evidence for these outcomes. Finally, the results analysed in this review were based on observational studies, and the reported associations cannot be translated into causation, especially as subclinical states of dementia can remain undiagnosed but affect diabetes management and glycaemic control.

## 5 CONCLUSION AND CLINICAL IMPLICATIONS

Hypoglycaemia, higher HbA1c levels, greater HbA1c variability and longer diabetes duration are all associated with an increased risk of all‐cause dementia and its subtypes in people with type 2 diabetes. This suggests that achieving and then maintaining target ranges of HbA1c while avoiding hypoglycaemia could help better manage dementia risk, especially in older individuals. Although the duration of diabetes is not a modifiable risk factor, its association with a greater risk of dementia highlights the need for good management of other dementia risk factors and considering dementia screening in individuals with longstanding diabetes.

## FUNDING INFORMATION

MT is supported by the La Trobe University Scholarships. JES and DJM are supported by National Health and Medical Research Council Investigator Fellowships.

## CONFLICT OF INTEREST STATEMENT

None.

## Supporting information


Data S1.
