# Supplementary material for: Associations of glycaemia‐related risk factors with dementia and cognitive decline in individuals with type 2 diabetes: A systematic review and meta‐analysis
Source: Diabet Med. 2025 Aug 19;42(10):e70123. doi: 10.1111/dme.70123 (PMC12434444; doi:10.1111/dme.70123)
Supplement: Supplementary file 1 — Data S1. [file DME-42-e70123-s001.docx]

# Supplementary Table 1. Search strategy used in MEDLINE and Embase

| Search line | Search terms | Results |
| --- | --- | --- |
| 1 | Diabetes Mellitus.sh. | 954476 |
| 2 | (Diabet* or T2DM).ti. | 1081018 |
| 3 | 1 or 2 | 1672897 |
| 4 | (Glycated Hemoglobin or risk factors).sh. | 1052805 |
| 5 | (Glycosylated Haemoglobin or Glycated Haemoglobin or Glycosylated Hemoglobin or Glycated Hemoglobin or HbA1c or glycemic control or glycaemic control or hypoglycemi* or hypoglycaemi* or risk factor or duration).tw. | 3012047 |
| 6 | 4 or 5 | 3858988 |
| 7 | (Cognitive Dysfunction or Dementia or Alzheimer or Alzheimer's Disease or Executive Function or Memory or Processing Speed).sh. | 579438 |
| 8 | (Cognitive Dysfunction or Cognitive Decline or Mild Cognitive Dysfunction or MIC or Dementia or Alzheimer or Alzheimer's Disease or Neurocognitive Disorder or Cognitive Function or Cognitive Impairment or Cognitive Performance or Executive Function or Memory or Systemic Neurodegenerative Disease or Major Neurocognitive Disorder).tw. | 1710105 |
| 9 | 7 or 8 | 1832305 |
| 10 | 3 and 6 and 9 | 10519 |
| 11 | observational.tw. | 786090 |
| 12 | longitudinal studies.sh. | 178016 |
| 13 | cohort studies.sh. | 350348 |
| 14 | prospective studies.sh. | 703432 |
| 15 | follow-up studies.sh. | 705814 |
| 16 | 11 or 12 or 13 or 14 or 15 | 2403190 |
| 17 | Pragmatic Clinical Trial/ or Clinical Trial, Phase III/ or Randomized Controlled Trial/ or Trial.mp. or Clinical Trial, Phase  II/ or "Trial of Labor"/ or Clinical Trial, Phase I/ or Clinical Trial, Phase IV/ or Clinical Trial/ or Controlled Clinical  Trial.mp. or case reports/ or clinical conference/ or clinical study/ [mp=ti, ab, hw, tn, ot, dm, mf, dv, kf, fx, dq, bt, nm, ox,  px, rx, ui, sy, ux, mx] | 7058241 |
| 18 | 17 not 16 | 6586241 |
| 19 | conference abstract.mp. [mp=ti, ab, hw, tn, ot, dm, mf, dv, kf, fx, dq, bt, nm, ox, px, rx, ui, sy, ux, mx] | 2416930 |
| 20 | letter*.mp. [mp=ti, ab, hw, tn, ot, dm, mf, dv, kf, fx, dq, bt, nm, ox, px, rx, ui, sy, ux, mx] | 2757657 |
| 21 | opinion*.mp. [mp=ti, ab, hw, tn, ot, dm, mf, dv, kf, fx, dq, bt, nm, ox, px, rx, ui, sy, ux, mx] | 378108 |
| 22 | editorial*.mp. [mp=ti, ab, hw, tn, ot, dm, mf, dv, kf, fx, dq, bt, nm, ox, px, rx, ui, sy, ux, mx] | 1616163 |
| 23 | Cross-Sectional Studies.sh. | 523327 |
| 24 | animal*.mp. [mp=ti, ab, hw, tn, ot, dm, mf, dv, kf, fx, dq, bt, nm, ox, px, rx, ui, sy, ux, mx] | 15375192 |
| 25 | protocol.mp. [mp=ti, ab, hw, tn, ot, dm, mf, dv, kf, fx, dq, bt, nm, ox, px, rx, ui, sy, ux, mx] | 1275232 |
| 26 | 18 or 19 or 20 or 21 or 22 or 23 or 24 or 25 | 28709228 |
| 27 | 10 not 26 | 6948 |
| 28 | covid.ti. | 612362 |
| 29 | coronavirus.ti. | 67579 |
| 30 | SARS-CoV-2.ti. | 144124 |
| 31 | 28 or 29 or 30 | 787524 |
| 32 | 27 not 31 | 6870 |
| 33 | limit 32 to english language | 6504 |
| 34 | limit 33 to yr="2000 -2024" | 6254 |
| 35 | limit 34 to "remove preprint records" | 6236 |

# Supplementary Table 2: Characteristics of included studies in each area of glycaemia-related risk factors

| Author and year | Country | Source of data | Study design | Study year | Diabetes sample size | Diabetes type | Average age (years) | Sex proportion (%male) | Average diabetes duration (years) | Average follow-up length (years) | Risk of bias score^a^ |
| --- | --- | --- | --- | --- | --- | --- | --- | --- | --- | --- | --- |
| Studies on association of hypoglycaemia with cognitive impairment/dementia | | | | | | | | | | | |
| Alkabbani 2023 | Canada | British Columbia health care data | cohort | 1996-2018 | 223,940 | T2DM | 69.9 ± 5.8 | 53.9 | 2.18 ± 3.71 | 6.7 | 4+2+3=9 |
| Chin 2016 | Korea | KNDP | cohort | 2006-2010 | 1,957 | T2DM | 67.5 ± 5.5 | 47.0 | 7.8 ±7.5 | 3.4 ± 0.9 | 4+2+3=9 |
| Feinkohl 2014 | Scotland | ET2DMS | cohort | 2006-2011 | 831 | T2DM | 67.69 ± 4.16 | 51.7 | 6.00 (3.00–11.00) | 4 | 2+2+3=7 |
| Haroon 2015 | Canada | Ontario provincial health administrative databases | cohort | 2007-2012 | 110,816 | All diabetes | 73 (69-78) | 49.2 | N/A | 7.2 | 4+2+3=9 |
| Karayiannides 2022 | Sweden | Sweden nationwide registries | cohort | 2013-2015 | 58,073 | T2DM | 77 | 60.3 | N/A | N/A | 4+2+3=9 |
| Kim 2020 | Korea | NHIS | cohort | 2002-2015 | 11,932 | T2DM | 75.82 ± 5.41 | 38.16 | N/A | 4.4 | 4+2+3=9 |
| Li 2022 | Taiwan | NHIRD | cohort | 2002-2011 | 677,618 | T2DM | 58.2 ± 12.0 | 49.08 | N/A | 6.7 | 4+2+3=9 |
| Lin 2013 | Taiwan | NHIRD | cohort | 1996-2009 | 15,404 | T2DM | 64.2 ± 9.9 | 45.1 | N/A | 3.8 | 4+2+3=9 |
| Mehta 2017 | UK | CPRD | cohort | 2003-2012 | 53,055 | T2DM | 75.0 ± 6.6 | 51.5 | N/A | 3.8 (1.8-6.3) | 3+2+3=8 |
| Whitmer 2009 | USA | KPNC Diabetes Registry | cohort | 2003-2007 | 16,667 | T2DM | 64.9 ± 7.1 | 54.6 | 9.6 ± 8.1 | 3.8 | 4+2+3=9 |
| Yaffe 2013 | USA | Health ABC | an analytic sample from a cohort study | 1997-2014 | 783 | All diabetes | 74.0 ± 2.8 | 52.4 | N/A | 12 | 4+2+2=8 |
| Zheng 2021 | UK | CPRD | cohort | 1987-2018 | 457,902 | T2DM | 64.5 ±10.8 | 52.1 | 0.6 | 6 | 4+2+3=9 |
| Cukierman-Yaffe 2019 | multi countries | ORIGIN trial | observational analysis of trial | 2003-2009 | 11,495 | T2DM | 63.3 ± 7.7 | 67.2 | N/A | 6.2 | 3+2+3=8 |
| Lee 2021 | Hong Kong | electronic health records of the Hong Kong Hospital Authority | cohort | 2008 - 2012 | 85,514 | T2DM | 71.3 ± 7.6 | 45.3 | 11.8 ± 7.7 | 6 | 4+2+3=9 |
| Gao 2024 | China | data collected by the authors | cohort | 2019-2022 | 234 | T2DM | 61.5 ± 7.3 | 72.1 | 7.6 | 3 | 4+2+3=9 |
| Han 2022 | Korea | National Health Insurance System in Korea | cohort | 2009-2015 | 2,032,689 | T2DM | 59.9 ± 10.6 | 57.7 | N/A | 6.9 ± 1.7 | 4+2+3=9 |
| Studies on association of HbA1c with cognitive impairment/dementia | | | | | | | | | | | |
| Beavers 2017 | USA | Look AHEAD M&M | observational analysis of trial | 2001-2012 | 879 | T2DM | 58.9 ± 6.8 | 43.6 | N/A | 7 | 3+2+3=8 |
| Celis-Morales 2022 | Sweden | Swedish NDR | cohort | 1998-2013 | 378,299 | T2DM | 64.13 ± 12.33 | 54.7 | 5.12 ± 6.79 | 7.03 | 4+2+3=9 |
| Cho 2023 | Korea | EMR database | cohort | 2005-2021 | 20,487 | T2DM | 71.7 ± 10.1 | 54.3 | N/A | 7.5 ± 3.4 | 4+2+3=9 |
| Christman 2011 | USA | ARIC Study | cohort | 1987-1998 | 516 | T2DM | 57.8 ± 5.6 | 44 | N/A | 14 | 4+2+2=8 |
| Feinkohl 2015 | Scotland | ET2DMS | cohort | 1988-2007 | 831 | T2DM | 67.7 ± 4.2 | 51.7 | 6 (3-11) | 4 | 3+2+3=8 |
| Li 2021 | UK | UK Biobank | cohort | 2006-2010 | 25,879 | T2DM | 59.9 ± 7.1 | 65.1 | N/A | 8.1 | 4+2+3=9 |
| Ma 2015 | China | data collected by the authors | cohort | 2010-2014 | 634 | T2DM | 75.81 ± 0.53 | 53.2 | N/A | 4.17 | 4+1+3=8 |
| Marden 2017 | USA | HRS | cohort | 2006-2012 | 8,888 | T2DM | 67.4 ± 8.8 | 46 | N/A | 5.2 | 4+0+3=7 |
| Mehta 2017 | UK | CPRD | cohort | 2003-2012 | 53,055 | T2DM | 75.0 ± 6.6 | 51.5 | N/A | 3.8 (1.8-6.3) | 3+2+3=8 |
| Pappas 2017 | USA | HRS | cohort | 2006-2012 | 950 | All diabetes | 72.57 ± 7.25 | 44.6 | N/A | > 6 | 4+2+2=8 |
| Wang 2022 | China | CHARLS | cohort | 2011-2018 | 935 | All diabetes | 60.39 ± 10.00 | 45.7 | N/A | 8 | 4+2+3=9 |
| Yaffe 2013 | USA | Health ABC | an analytic sample from a cohort study | 2002-2011 | 783 | All diabetes | 74.0 ± 2.8 | 52.4 | N/A | 12 | 4+2+2=8 |
| Yu 2022 | Korea | KoGES | cohort | 2001-2018 | 765 | T2DM | 60.6 ± 6.9 | 55.6 | 7.9 (3.9-9.8) | 4.2 ± 0.5 | 4+2+3=9 |
| Zaslavsky 2020 | USA | ACT study | cohort |  | 316 | All diabetes | 83 ± 3 | 40.49 | N/A | N/A | 4+1+2=7 |
| Zhao 2020 | China | data collected by the authors | cohort |  | 1,519 | T2DM | 72 ± 6.3 | 38.2 | N/A | 3 | 4+2+2=8 |
| Rawlings 2019 | USA | ARIC Study | cohort |  | 1,735 | All diabetes | 75.8 ± 5.0 | 42.5 | N/A | 5 | 4+2+3=9 |
| Zheng 2021 | UK | CPRD | cohort |  | 457,902 | T2DM | 64.5 | 52.1 | 0.6 | 6 | 4+2+3=9 |
| Lee 2021 | Hong Kong | electronic health records of the Hong Kong Hospital Authority | cohort | 2008 - 2012 | 85,514 | T2DM | 71.3 ± 7.6 | 45.3 | 11.8 ± 7.7 | 6 | 4+2+3=9 |
| Umegaki 2012 | Japan | J-EDIT | observational analysis of trial | 2001-2007 | 261 | T2DM | 70.6 ± 4.3 | 42.5 | N/A | 6 | 2+1+2=5 |
| Gao 2024 | China | data collected by the authors | cohort | 2019-2022 | 234 | T2DM | 61.5 ± 7.3 | 72.1 | 7.6 | 3 | 4+2+3=9 |
| Underwood 2024 | USA | Veterans’ Health Administration and Medicare | cohort | 2004-2018 | 374,021 | All diabetes | 73.2 ± 5.8 | 99 | N/A | Up to 10 | 4+2+3=9 |
| O 2023 | Hong Kong | Hong Kong Diabetes Register | cohort | 2010-2015 | 986 | T2DM | 62.5 ±2.6 | 58.3 | 10.7 ± 8.2 | 7.6 | 4+2+3=9 |
| Studies on association of HbA1c variability with cognitive impairment/dementia | | | | | | | | | | | |
| Yu 2020 | USA and England | HRS and ELSA | pooled analysis of two cohorts | HRS: 2006 - 2016, ELSA: 2002 - 2015 | 651 | All diabetes | 63.38 ± 8.26 | 41.8 | N/A | HRS: 10.48 ± 0.63 ; ELSA: 10.56 ± 1.86 | 4+2+3=9 |
| Zheng 2021 | UK | CPRD | cohort | 1987-2018 | 457,902 | T2DM | 64.5 | 52.1 | 0.6 | 6 | 4+2+3=9 |
| Li 2017 | Taiwan | National Diabetes Care Management Program | cohort | 2002-2011 | 16,706 | T2DM | 62.2 ± 6.0 | 45.12 | 8.04 ± 7.28 | 8.8 | 4+2+3=9 |
| Lee 2021 | Hong Kong | electronic health records of the Hong Kong Hospital Authority | cohort | 2008 - 2012 | 85,514 | T2DM | 71.3 ± 7.6 | 45.3 | 11.8 ± 7.7 | 6 | 4+2+3=9 |
| Moran 2024 | USA | KPNC | cohort | 1996 - 2018 | 171,964 | T2DM | 61.1 ± 9.1 | 52 | N/A | 5.1 | 4+2+3=9 |
| Studies on association of duration of diabetes with cognitive impairment/dementia | | | | | | | | | | | |
| Bruce 2019 | Australia | Fremantle Diabetes Study Phase 1 | cohort | 1993 - 2012 | 1,291 | T2DM | 64 ± 11.3 | 48.6 | N/A | 12.7 ± 5.9 | 4+2+3=9 |
| Parikh 2011 | USA | inpatient and outpatient records for Veterans Administration | cohort | 1996 - 2000 | 377,838 | All diabetes | 75.53 ± 6.07 | 97.8 | N/A | 2 | 3+1+2=6 |
| Reinke 2022 | Germany | AOK | cohort | 2006 - 2014 | 13,761 | T2DM | 76.9 ± 5.8 | 39.2 | 5 (2.3–8.5) | 4.18 | 4+2+3=9 |
| Yu 2020 | Korea | NHIS | cohort | 2009 - 2015 | 1,917,702 | T2DM | 59.73 ± 10.56 | 57.9 | groups based on duration of diabetes: <5 y:  1.0 (0.0–3.0)  5 to 10 y:  7.0 (5.0–8.0)  10 to 15 y:  11.0 (10.0–13.0)  ≥15 y:  23.0 (18.0–36.0) | 5.1 | 4+2+3=9 |
| Lee 2021 | Hong Kong | electronic health records of the Hong Kong Hospital Authority | cohort | 2008 - 2012 | 85,514 | T2DM | 71.3 ± 7.6 | 45.3 | 5.6 ± 5.1 | 6 | 4+2+3=9 |
| Li 2021 | UK | UK Biobank | cohort | 2006-2010 | 25,879 | T2DM | 59.9 ± 7.1 | 65.1 | 4.0 (1.0–8.0) | 8.1 | 4+2+3=9 |
| Ma 2015 | China | data collected by the authors | cohort | 2010-2014 | 634 | T2DM | 75.81 ± 0.53 | 53.2 | 5.0 (1.0–11.0) | 4.17 | 4+1+3=8 |
| Rawlings 2019 | USA | ARIC Study | cohort | 1993 - 2012 | 1,735 | All diabetes | 75.8 ± 5.0 | 42.5 | <5: 48.7%  5–<10: 27.1%  10–<15: 18.5%  ≥ 15: 5.7 | 5 | 4+2+3=9 |

^a^ Risk of bias is reported as Selection + Comparability + Outcome = Overall score

Abbreviations:

ACT: Adult Changes in Thought study, AOK: German health insurance Allgemeine Ortskrankenkasse, ARIC: Atherosclerosis Risk in Communities Study, CHARLS: China Health and Retirement Longitudinal Study, CPRD: Clinical Practice Research Datalink, ELSA: English Longitudinal Study of Aging, EMR: electronic medical records database, ET2DMS: Edinburgh Type 2 Diabetes Study, Health ABC: Health, Aging, and Body Composition Study, HRS: The Health Retirement Study, KNDP: Korea National Diabetes Program, KoGES: Ansan cohort of Korean Genome Epidemiology Study, KPNC: Kaiser Permanente Northen California, Look AHEAD M&M: Action for Health in Diabetes Movement and Memory, NDR: National Diabetes Register, NHIRD: Taiwan National Health Insurance Research Database, NHIS: National Korean Health Insurance Service, ORIGIN: Outcome Reduction with an Initial Glargine Intervention Trial, T2DM: type 2 diabetes mellitus, UK: the United Kingdom, USA: the United States of America

# Supplementary Table 3: Reported effect sizes for the association of glycaemia-related risk factors with decline in cognitive function and dementia

| Author and year | Effect size (95% CI)^a^ | |
| --- | --- | --- |
| Studies on association of hypoglycaemia with cognitive impairment/dementia^b^ | |
| Alkabbani 2023 | Any episode: 2.63 (2.01, 3.45) | |
| Chin 2016 | Minimally adjusted model (adjusted for age, sex, BMI, diabetes duration, smoking and alcohol status and diastolic blood pressure):  Any episode: 2.03 (0.89, 4.62)  Dose response association:  One episode: 1.51 (0.47, 4.90)  Two or more episodes: 2.83 (0.96, 8.35)  Maximally adjusted model:  Any episode: 2.69 (1.08, 6.69)  Dose response association:  One episode: 2.05 (0.61, 6.91)  Two or more episodes: 4.07 (1.10, 15.04) | |
| Feinkohl 2014 | Lower *g* at four-year follow-up (OR reported): 1.65 (0.99, 2.76)  Steeper decline in *g* between baseline and follow-up (OR reported): 1.36 (0.82, 2.24) | |
| Haroon 2015 | Any episode: 1.73 (1.62, 1.84) | |
| Karayiannides 2022 | Any episodes: 1.37 (1.08, 1.73) | |
| Kim 2020 | Any episode:  All-cause dementia: 1.25 (1.17, 1.35)  Alzheimer’s disease: 1.26 (1.16, 1.38)  Vascular dementia: 1.29 (1.11, 1.49)  Dose response association, all-cause dementia:  One episode: 1.17 (1.04, 1.31)  Two or three episodes: 1.20 (1.02, 1.42)  More than three episodes: 1.36 (1.06, 1.74) | |
| Li 2022 | Stratified risk based on trajectories of hypoglycaemia episodes after enrolment (sub-distribution HR reported):  Group 1 (very late manifestation): 0.94 (0.84, 1.06)  Group 2 (late manifestation): 1.01 (0.93, 1.10)  Group 3 (early manifestation but with later decrease): 1.22 (1.14, 1.31)  Group 4 (early and sustained manifestation): 1.25 (1.02, 1.54)  We calculated a pooled sub-distribution HR using a fixed-effect model: 1.10 (1.05, 1.16) | |
| Lin 2013 | Minimally adjusted model (adjusted for age and sex):  Any episodes: 1.60 (1.19, 2.14)  Dose response association:  One episode: 1.47 (1.04, 2.07)  Two episodes: 1.41 (0.73, 2.71)  Three or more episodes: 2.60 (0.97, 6.92)  Maximally adjusted model:  Any episodes: 1.45 (1.07, 1.95), 0.015 | |
| Mehta 2017 | Minimally adjusted model (adjusted for age, gender and baseline): 1.35 (1.14, 1.61)  Maximally adjusted model: 1.27 (1.06, 1.51) | |
| Whitmer 2009 | Minimally adjusted model (adjusted for age, sex, race, education, BMI and diabetes duration):  Any episodes: 1.68 (1.47, 1.93)  Dose response association:  One episode: 1.45 (1.23, 1.72)  Two episodes: 2.15 (1.64, 2.81)  Three or more episodes: 2.60 (1.78, 3.79)  Maximally adjusted model:  Any episodes: 1.44 (1.25, 1.66)  Dose response association:  One episode: 1.26 (1.10, 1.49)  Two episodes: 1.80 (1.37, 2.36)  Three or more episodes: 1.94 (1.42, 2.64) | |
| Yaffe 2013 | Any episodes: 2.09 (1.00, 4.35) | |
| Zheng 2021 | Minimally adjusted model (adjusted for age, sex, calendar year, region): 1.50 (1.42, 1.59)  Maximally adjusted model: 1.30 (1.22, 1.39) | |
| Cukierman-Yaffe 2019 | Any episodes: 1.04 (0.79, 1.38) | |
| Lee 2021 | Any episodes, Alzheimer's disease:  Women: 1.69 (1.14, 2.52)  Men: 0.93 (0.42, 2.90) | |
| Gao 2024 | Mild cognitive impairment, reporting OR for having more than five episodes of hypoglycaemia:  ≤5 episodes: reference  >5 episodes: 4.64 (1.28, 16.78) | |
| Han 2022 | Minimally adjusted model (adjusted for age, sex):  All-cause dementia:  One episode: 1.88 (1.81, 1.95)  Two or more episodes: 2.36 (2.19, 2.55)  Alzheimer’s disease:  One episode: 1.83 (1.75, 1.92)  Two or more episodes: 2.41 (2.21, 2.64)  Vascular dementia:  One episode: 1.91 (1.71, 2.13)  Two or more episodes: 1.97 (1.54, 2.52)  Maximally adjusted model:  All-cause dementia:  One episode: 1.54 (1.48, 1.60)  Two or more episodes: 1.80 (1.66, 1.94)  Alzheimer’s disease:  One episode: 1.52 (1.45, 1.59)  Two or more episodes: 1.87 (1.71, 2.04)  Vascular dementia:  One episode: 1.50 (1.34, 1.67)  Two or more episodes: 1.43 (1.12, 1.83) | |
| Studies on association of HbA1c with cognitive impairment/dementia^b^ | |
| Beavers 2017 | Minimally adjusted model (b Estimate reported):  Trail-Making Test, Part A: 0.05 (0.01, 0.11)  Trail-Making Test, Part B: 0.00 (0.06, 0.06)  Modified Stroop Colour Word Test: 0.08 (0.78, 0.94)  Digit Symbol Coding: 0.05 (0.11, 0.01)  Rey Auditory Verbal Learning Test, short-delayed recall: -0.03 (0.09, 0.03)  Rey Auditory Verbal Learning Test, long-delayed recall: -0.04 (0.10, 0.02)  Modified Mini-Mental State Examination: 0.02 (0.04, 0.08)  Maximally adjusted model (b Estimate reported):  Trail-Making Test, Part A: 0.02 (-0.04, 0.08)  Trail-Making Test, Part B: -0.02 (-0.08, 0.04)  Modified Stroop Colour Word Test: 0.08 (0.78, 0.94)  Digit Symbol Coding: -0.02 (-0.07, 0.04)  Rey Auditory Verbal Learning Test, short-delayed recall: -0.03 (-0.10, 0.03)  Rey Auditory Verbal Learning Test, long-delayed recall: -0.05 (-0.11, 0.01)  Modified Mini-Mental State Examination: 0.03 (0.03, 0.09) | |
| Celis-Morales 2022 | Alzheimer's disease:  Per 1 mmol/mol increase in HbA1c: 1.004 (1.001, 1.008) (calculated HR per percentage point increase in HbA1c: 1.05 (1.01, 1.09)  Categorical association of HbA1c:  <53 mmol/mol (7%): reference  53–64 mmol/mol (7–8%): 1.02 (0.92, 1.12)  65–75 mmol/mol (8.1–9%): 1.08 (0.94, 1.25)  76–86 mmol/mol (9.1–10%): 1.32 (1.06, 1.65)  ≥87 mmol/mol (10%): 1.35 (1.04, 1.75)  Vascular dementia:  Per 1 mmol/mol increase in HbA1c: 1.01 (1.01, 1.02) (calculated HR per percentage point increase in HbA1c: 1.15 (1.12, 1.24)  Categorical association of HbA1c:  <53 mmol/mol (7%): reference  53–64 mmol/mol (7–8%): 1.18 (1.07, 1.31)  65–75 mmol/mol (8.1–9%): 1.40 (1.22, 1.61)  76–86 mmol/mol (9.1–10%): 1.75 (1.43, 2.14)  ≥87 mmol/mol (10%): 1.93 (1.54, 2.43)  Nonvascular dementia:  Per 1 mmol/mol increase in HbA1c: 1.009 (1.007, 1.011)  Categorical association of HbA1c:  <53 mmol/mol (7%): reference  53–64 mmol/mol (7–8%): 1.10 (1.04, 1.16)  65–75 mmol/mol (8.1–9%): 1.32 (1.22, 1.42)  76–86 mmol/mol (9.1–10%): 1.46 (1.30, 1.65)  ≥87 mmol/mol (10%): 1.67 (1.46, 1.91) | |
| Cho 2023 | All-cause dementia:  Per 1 percentage point increase in HbA1c: 1.43 (1.35, 1.51)  Categorical association of HbA1c:  <53 mmol/mol (7%): reference  53–64 mmol/mol (7–8%): 1.28 (1.04, 1.55)  64–75 mmol/mol (8–9%): 1.79 (1.24, 2.58)  75–86 mmol/mol (9–10%): 2.81 (1.57, 5.05)  ≥86 mmol/mol (10%): 1.67 (1.46, 1.91)  Alzheimer’s disease:  Per 1 percentage point increase in HbA1c: 1.40 (1.31, 1.51) | |
| Christman 2011 | Categorical association of HbA1c:  <53 mmol/mol (7%): reference  53–64 mmol/mol (7–8%): 0.97 (0.37, 2.55)  >64 mmol/mol (8%): 1.13 (0.43, 2.98) | |
| Feinkohl 2015 | Lower *g* at four-year follow-up (OR reported): 1.24 (1.02, 1.49)  Steeper decline in *g* between baseline and follow-up (OR reported): 1.21 (1.00, 1.45) | |
| Li 2021 | Minimally adjusted model (age and sex adjusted):  All-cause dementia:  <53 mmol/mol (7%): reference  53–58 mmol/mol (7–7.5%): 1.06 (0.78, 1.45)  59–63 mmol/mol (7.5–8%): 1.23 (0.85, 1.78)  ≥64 mmol/mol (10%): 1.42 (1.06, 1.89)  Alzheimer’s disease:  <53 mmol/mol (7%): reference  53–58 mmol/mol (7–7.5%): 0.71 (0.37, 1.34)  59–63 mmol/mol (7.5–8%): 1.14 (0.58, 2.22)  ≥64 mmol/mol (10%): 1.75 (1.10, 2.79)  Vascular dementia:  <53 mmol/mol (7%): reference  53–58 mmol/mol (7–7.5%): 2.08 (1.32, 3.29)  59–63 mmol/mol (7.5–8%): 1.71 (0.93, 3.14)  ≥64 mmol/mol (10%): 1.98 (1.23, 3.19)  Maximally adjusted model:  All-cause dementia:  <53 mmol/mol (7%): reference  53–58 mmol/mol (7–7.5%): 1.02 (0.75, 1.39)  59–63 mmol/mol (7.5–8%): 1.05 (0.72, 1.52)  ≥64 mmol/mol (10%): 1.18 (0.88, 1.59)  Alzheimer’s disease:  <53 mmol/mol (7%): reference  53–58 mmol/mol (7–7.5%): 0.70 (0.37, 1.34)  59–63 mmol/mol (7.5–8%): 1.00 (0.51, 1.96)  ≥64 mmol/mol (10%): 1.56 (0.96, 2.52)  Vascular dementia:  <53 mmol/mol (7%): reference  53–58 mmol/mol (7–7.5%): 1.97 (1.24, 3.12)  59–63 mmol/mol (7.5–8%): 1.44 (0.78, 2.67)  ≥64 mmol/mol (10%): 1.68 (1.03, 2.74) | |
| Ma 2015 | Categorical association of HbA1c:  <53 mmol/mol (7%): reference  ≥53 mmol/mol (7%): 1.298 (1.105, 1.573) | |
| Marden 2017 | Reporting b estimates for memory decline per percentage point increase in HbA1c: -0.011 (-0.022, 0.0003) | |
| Mehta 2017 | Minimally adjusted model (adjusted for age, gender and baseline):  Categorical association of HbA1c:  27–41 mmol/mol (2.5 –5.9%): 1.23 (1.03, 1.46)  42–52 mmol/mol (6–6.9%): 1.01 (0.92, 1.11)  53–63 mmol/mol (7–7.9%): reference  64–74 mmol/mol (8–8.9%): 0.98 (0.86, 1.10)  ≥75 mmol/mol (9%): 0.96 (0.86, 1.07)  Maximally adjusted model:  Categorical association of HbA1c:  27–41 mmol/mol (2.5 –5.9%): 1.21 (1.01, 1.44)  42–52 mmol/mol (6–6.9%): 1.01 (0.92, 1.12)  53–63 mmol/mol (7–7.9%): reference  64–74 mmol/mol (8–8.9%): 0.96 (0.85, 1.08)  ≥75 mmol/mol (9%): 0.94 (0.84, 1.05) | |
| Pappas 2017 | Reporting b for decline in episodic memory: 0.05 (-0.40, 0.50) | |
| Wang 2022 | Reporting b for change in general cognition functioning per percentage point increase in HbA1c: -0.05 (-0.36, 0.27) | |
| Yaffe 2013 | Per 1 percentage point increase in HbA1c: 1.00 (0.85-1.17) | |
| Yu 2022 | Reporting β (95% CI) changes in different domains of cognitive function:  executive function Z score:  Baseline HbA1c: -0.653 (-1.073, -0.234)  Time-weighted HbA1c: -0.818 (-1.290, -0.346)  global cognitive Z score:  Baseline HbA1c: 0.430 (-0.322, 1.182)  Time-weighted HbA1c: -0.486 (-0.936, -0.036) | |
| Zaslavsky 2020 | Reporting beta estimates for difference in Cognitive Ability Screening Instrument Item Response Theory score comparing categories of HbA1c:  53–64 mmol/mol (7–7.9%): reference  <53 mmol/mol (7%):  age 80: -0.18 (-0.35, -0.02)  age 84: -0.06 (-0.17, 0.05)  age 88: 0.06 (-0.07, 0.20)  age 92: 0.19 (-0.03, 0.40)  >64 mmol/mol (8%):  age 80: -0.22 (-0.40, − 0.05)  age 84: -0.12 (-0.24, -0.01)  age 88: -0.02 (-0.17, 0.13)  age 92: 0.08 (-0.16, 0.32) | |
| Zhao 2020 | Reporting beta (95% CI) for decline in MMSE score comparing categories of HbA1c:  53–63 mmol/mol (7–7.9%): reference  <53 mmol/mol (7%): 0.05 (-0.37, 0.47)  ≥64 mmol/mol (8%): 0.58 (0.06, 1.11) | |
| Rawlings 2019 | Categorical association of HbA1c:  <53 mmol/mol (7%): reference  ≥53 mmol/mol (7%): 1.15 (0.75, 1.77) | |
| Zheng 2021 | Minimally adjusted model (adjusted for age, sex, calendar year and region):  Per 1 percentage point increase in HbA1c: 1.13 (1.12, 1.14)  Categorical association of HbA1c:  <42 mmol/mol (6%): 0.86 (0.83, 0.89)  42–53 mmol/mol (6–7%): reference  53–64 mmol/mol (7–8%): 1.02 (0.98 - 1.06)  64–75 mmol/mol (8–9%): 1.15 (1.09 - 1.21)  75–86 mmol/mol (9–10%): 2.81 1.26 (1.17 - 1.34)  ≥86 mmol/mol (10%): 1.40 (1.32 - 1.49)  Maximally adjusted model (adjusted for age, sex, calendar year and region):  Per 1 percentage point increase in HbA1c: 1.08 (1.07–1.09) | |
| Lee 2021 | Alzheimer's disease:  Women: 0.95 (0.90, 1.02)  Men: 1.08 (1.00, 1.17) | |
| Umegaki 2012 | Reporting OR for decline in MMSE score: 1.03 (0.62, 1.72) | |
| Gao 2024 | Reporting OR for decline in MoCA score: 1.663 (0.911–3.037) | |
| Underwood 2024 | Reporting HR comparing categories of HbA1c time in range (TIR):  80% to 100%: reference  60% to <80%: 1.07 (1.03, 1.11)  40% to <60%: 1.06 (1.02, 1.10)  20% to <40%: 1.14 (1.09, 1.18)  0 to <20%: 1.19 (1.16, 1.23) | |
| O 2023 | Categorical association of HbA1c:  <53 mmol/mol (7%): reference  ≥53 mmol/mol (7%): 2.245 (1.045, 4.824) | |
| Studies on association of HbA1c variability with cognitive impairment/dementia^b^ | |
| Yu 2020 | Reporting β (95% CI) for association of each SD increment in the CV of HbA1c with cognitive decline:  Memory z score: -0.028 (-0.065, 0.008)  Executive function z score: 0.018 (-0.029, 0.064) | |
| Zheng 2021 | Reporting three-year CV of HbA1c (per 1 SD):  Minimally adjusted model (adjusted for age, sex, calendar year, region): 1.02 (1.00, 1.04)  Maximally adjusted model: 1.03 (1.01, 1.04) | |
| Li 2017 | Reporting HR for Alzheimer's disease according to tertiles of HbA1c CV:  Minimally adjusted model (adjusted for age and sex):  ≤ 8.3%: reference  8.3–16.3%: 1.08 (0.90, 1.28)  >16.3%: 1.60 (1.35, 1.89)  Maximally adjusted model:  ≤ 8.3%: reference  8.3–16.3%: 0.98 (0.82, 1.17)  >16.3: 1.32 (1.11, 1.58) | |
| Lee 2021 | HbA1c% SD:  Women: 1.14 (1.02, 1.27)  Men: 1.09 (0.94, 1.26)  HbA1c% CV:  Women: 1.01 (1.00, 1.02)  Men: 1.01 (0.99, 1.02) | |
| Moran 2024 | Minimally adjusted model (adjusted for age, sex, race, ethnicity):  SD: 1.15 (1.12, 1.17)  CV: 2.56 (2.14, 3.06)  ARV: 1.10 (1.09, 1.11)  Maximally adjusted model:  SD: 1.15 (1.12, 1.17)  CV: 2.54 (2.12, 3.05)  ARV: 1.10 (1.09, 1.11) | |
| Studies on association of duration of diabetes with cognitive impairment/dementia^b^ | |
| Bruce 2019 | Per 1 year increment: 1.02 (1.002, 1.04) | |
| Parikh 2011 | Categorical association:  1 year: reference  2 years: 1.05 (0.99, 1.11)  3 years: 1.12 (1.05, 1.18)  4 years: 1.33 (1.26, 1.40)  5 years or more: 1.43 (1.36, 1.50) | |
| Reinke 2022 | Reporting HRs from a piecewise exponential regression model:  Minimally adjusted model (adjusted for age, sex, comorbidity and complication severity index):  0.918 (0.904 - 0.932) for linear term  1.002 (1.002, 1.003) for quadratic term  Maximally adjusted model:  0.899 (0.864, 0.935) for linear term  1.003 (1.001, 1.004) for quadratic term | |
| Yu 2020 | All-cause dementia:  <5 years: reference  >5 years: 1.31 (1.29, 1.33)  Alzheimer's disease:  <5 years: reference  >5 years: 1.30 (1.28, 1.32)  Vascular dementia:  <5 years: reference  >5 years: 1.32 (1.28, 1.37) | |
| Lee 2021 | Reporting hazard ratio per 1 year increment in diabetes duration:  Women: 1.09 (1.03, 1.15)  Men: 1.21 (1.11, 1.31) | |
| Li 2021 | Minimally adjusted model (adjusted for age and sex):  All-cause dementia:  <5 years: reference  5–10 years: 1.58 (1.20, 2.09)  10–15 years: 2.02 (1.44, 2.83)  >15 years: 2.76 (2.08, 3.65)  Alzheimer’s disease:  <5 years: reference  5–10 years: 1.64 (1.02, 2.64)  10–15 years: 1.82 (0.99, 3.34)  >15 years: 2.39 (1.44, 3.97)  Vascular dementia:  <5 years: reference  5–10 years: 1.59 (0.97, 2.61)  10–15 years: 2.81 (1.65, 4.80)  >15 years: 3.39 (2.12, 5.42)  Maximally adjusted model:  All-cause dementia:  <5 years: reference  5–10 years: 1.48 (1.12, 1.97)  10–15 years: 1.71 (1.21, 2.41)  >15 years: 2.15 (1.60, 2.90)  Alzheimer’s disease:  <5 years: reference  5–10 years: 1.49 (0.92, 2.42)  10–15 years: 1.37 (0.73, 2.55)  >15 years: 1.59 (1.02, 2.11)  Vascular dementia:  <5 years: reference  5–10 years: 1.44 (0.88, 2.38)  10–15 years: 2.25 (1.30, 3.89)  >15 years: 2.50 (1.53, 4.09) | |
| Ma 2015 | Categorical association:  1 year: reference  2 years: 1.04 (0.98, 1.10)  3 years: 1.10 (1.04, 1.17)  4 years: 1.32 (1.25, 1.39)  5 years or more: 1.42 (1.35, 1.49) | |
| Rawlings 2019 | Categorical association:  <5 years: reference  ≥5 years: 1.91 (1.09, 3.35) | |

^a^ The reported effect sizes referred to hazard ratio unless specified otherwise.

^b^ The outcome was all-cause dementia unless specified otherwise.

Abbreviations and notations: ARV: Average real variability; CI: confidence interval; CV: coefficient of variation; HR: hazard ratio; MMSE: Mini-Mental State Examination; MoCA: Montreal Cognitive Assessment; OR: odds ratio; SD: standard deviation; β: standardised beta coefficient

# Supplementary Table 4: Definitions of hypoglycaemia in included studies

| Study | Definition of hypoglycaemia |
| --- | --- |
| Alkabbani 2023 | Severe hypoglycaemic was defined as occurrence of one or more episodes based on following codes indicating hospitalisation or a physician claim for hypoglycaemia. ICD-9 codes: 251.0 (Hypoglycaemic coma), 251.1 (Other specified hypoglycaemia), 251.2 (Hypoglycaemia, unspecified), 251.0 (Hypoglycaemic coma), 251.1 (Other specified hypoglycaemia), 251.2 (Hypoglycaemia, unspecified); ICD-10 codes: E16.0 (Drug-induced hypoglycaemia), E16.1 (Other hypoglycaemia), E16.2 (Hypoglycaemia, unspecified), E15 (Non-diabetic hypoglycaemia), E11.63 (Type 2 diabetes with hypoglycaemia), E13.63 (Other specified diabetes with hypoglycaemia), E14.63 (Unspecified diabetes with hypoglycaemia), E16.0 (Drug-induced hypoglycaemia), E16.1 (Other hypoglycaemia), E16.2 (Hypoglycaemia, unspecified) |
| Chin 2016 | Hypoglycaemia was defined with the following diagnostic codes in the HIRAS claim database: E11.63, non-insulin-dependent diabetes mellitus (DM) with hypoglycaemia; E12.63, malnutrition-related DM with hypoglycaemia; E13.63, other specified type of DM with hypoglycaemia; E14.63, unspecified DM with hypoglycaemia; E16.0, drug-induced hypoglycaemia without coma; E16.1, other type of hypoglycaemia; and E16.2, unspecified hypoglycaemia. Information was unavailable regarding episodes of severe hypoglycaemia which required hospital admission. |
| Feinkohl 2014 | Self-reported history of severe hypoglycaemia defined as an episode requiring the assistance of another person to effect recovery plus questionnaires based on the Edinburgh Hypoglycaemia scale that comprised items on symptoms, date and time of any hypoglycemics episode, loss of consciousness, help from another person, treatment, and blood glucose values, if measured. |
| Haroon 2015 | Hypoglycaemia was defined as hospital admissions or emergency department visits for hypoglycaemia based on provincial health administrative databases in Ontario, Canada. |
| Karayiannides 2022 | History of severe hypoglycaemia was defined as a primary or secondary hospital ICD-10 diagnosis of E16.0, E16.1, E16.2, E10.0, E11.0, E12.0, E13.0, or E14.0. |
| Kim 2020 | History of hypoglycaemia defined as ICD-10 codes E11.63, E15, E16.0, E16.1, and E16.2 in Korean National Health Insurance Service. |
| Li 2022 | Severe hypoglycaemia was defined as ICD-9-CM codes 250.8, 251.0, 251.1, 251.2, 270.3, and 962.3 recorded in medical claims from emergency or inpatient departments. |
| Lin 2013 | Hypoglycaemia was defined as ICD-9-CM codes 251.0 (hypoglycaemic coma), 251.1 (other specified hypoglycaemia) and 251.2 (hypoglycaemia, unspecified) from the outpatient or inpatient databases in National Health Insurance of Taiwan. |
| Mehta 2017 | Hypoglycaemia was defined based on general practice records in CPRD containing codes for severe and non-severe hypoglycaemic episodes. |
| Whitmer 2009 | Hypoglycaemia defined based on hospitalisation and emergency department diagnostic ICD-9-CM codes 251.0 (hypoglycaemic coma), 251.1 (other specified hypoglycaemia), and 251.2(hypoglycaemia, unspecified). |
| Yaffe 2013 | Severe hypoglycaemia identified as the primary or secondary diagnosis related to any overnight hospitalisation in acute care hospital records. Episodes of mild hypoglycaemia were not included. |
| Zheng 2021 | Episodes of hypoglycaemia were defined using the following CPRD Medcodes: 52409 (Other hypoglycaemia), 32885 (Drug-induced hypoglycaemia without coma), 40363 (Frequency of GP or paramedic treated hypoglycaemia), 31752 (Frequency of hospital treated hypoglycaemia), 13281 (Frequency of hypoglycaemic Attacks), 1410 (Hypoglycaemia unspecified), 20368 (Hypoglycaemia unspecified NOS), 18583 (Hypoglycaemic attack requiring third party assistance), 2472 (Hypoglycaemic coma), 51371 (Hypoglycaemic coma NOS), 13068 (Last hypoglycaemic Attack), 43785 (Non-insulin dependent diabetes mellitus with hypoglycaemic coma), 95920 (Other hypoglycaemia), 68960 (Polyneuropathy in hypoglycaemia), 96942 (Post-prandial hypoglycaemia), 4563 (Reactive hypoglycaemia NOS), 37625 (Recurrent severe hypoglycaemia), 24405 (Spontaneous hypoglycaemia NOS), 61071 (Type 2 diabetes mellitus with hypoglycaemic coma), 46917 (Type 2 diabetes mellitus with hypoglycaemic coma), 56268 (Type II diabetes mellitus with hypoglycaemic coma), 98723 (Type II diabetes mellitus with hypoglycaemic coma) |
| Cukierman-Yaffe 2019 | Severe hypoglycaemia was defined as a symptomatic event requiring the assistance of another person with prompt recovery after carbohydrate/glucagon administration and/or a measured plasma glucose level of ≤36 mg/dL (2 mmol/L). |
| Lee 2021 | Severe hypoglycaemia defined as episodes requiring hospital attendance or admission in Hong Kong Hospital Authority electronic records. |
| Gao 2024 | Hypoglycaemia was defined as a blood glucose level below 70 mg/dL (3.9 mmol/ L) or an episode characterised by physical and/or mental functioning alteration requiring assistance from others. |
| Han 2022 | Severe hypoglycaemia was defined as any events requiring the assistance of another person to actively administer carbohydrates, or the need for corrective action, or adjustment of medical treatment, or hospitalisation (ICD-10 codes E16.x, E11.63, E13.63, and E14.63 in inpatient and emergency room claims). |

# Supplementary Table 5. Summary of results from studies evaluating the association of HbA1c level with measures of cognitive function in people with type 2 diabetes

| Domain | Test | Study | HbA1c categories^a^ | Subgroup | Results^b^ |
| --- | --- | --- | --- | --- | --- |
| Global cognition | Modified MMSE (standardised score) | Beavers, 2017 |  |  | 0.02 (-0.04, 0.08) |
|  | MMSE (decline in score) | Zhao, 2020 | 7%–8% (53–64 mmol/mol) |  | Reference |
|  |  |  | <7% (53 mmol/mol) |  | 0.05 (-0.37, 0.47)^c^ |
|  |  |  | >8% (64 mmol/mol) |  | 0.58 (0.06, 1.11)^c^ |
|  | MMSE (decline in score) | Umegaki, 2012 |  |  | 1.03(0.62, 1.72)^c, d^ |
|  | MoCA (decline in score) | Gao, 2024 |  |  | 1.66 (0.91, 3.04)^c, d^ |
|  | CASI-IRT | Zaslavsky, 2020 | 7%–8% (53–64 mmol/mol) |  | Reference |
|  |  |  | <7% (53 mmol/mol) | age 80 | -0.18 (-0.35, -0.02) |
|  |  |  |  | age 84 | -0.06 (-0.17, 0.05) |
|  |  |  |  | age 88 | 0.06 (-0.07, 0.20) |
|  |  |  |  | age 92 | 0.19 (-0.03, 0.40) |
|  |  |  | >8% (64 mmol/mol) | age 80 | -0.22 (-0.40, -0.05) |
|  |  |  |  | age 84 | -0.12 (-0.24, -0.01) |
|  |  |  |  | age 88 | -0.02 (-0.17, 0.13) |
|  |  |  |  | age 92 | 0.08 (-0.16, 0.32) |
|  | global cognitive performance (*g*)^e^ | Feinkohl, 2015 |  |  | 1.24 (1.02, 1.49)^d, c^ |
|  | general cognition functioning | Wang, 2022 |  |  | -0.05 (-0.36, 0.27) |
|  | global cognitive (standardised score) | Yu, 2022 |  |  | 0.43 (-0.32, 1.18)^f^ |
|  |  |  |  |  | -0.49 (-0.94, -0.04)^g^ |
| Episodic memory | RAVLT, short-delayed recall (standardised score) | Beavers, 2017 |  |  | -0.03 (-0.09, 0.03) |
|  | RAVLT, long-delayed recall (standardised score) | Beavers, 2017 |  |  | -0.04 (-0.10, 0.02) |
|  | combined proxy and direct memory assessments (decline in memory) | Marden, 2017 |  |  | -0.01 (-0.02, 0.00)^c^ |
|  | combined immediate and delayed word recall test (standardised score) | Pappas, 2017 |  |  | 0.05 (-0.40, 0.50) |
| Simple attention | TMT-A (standardised score) | Beavers, 2017 |  |  | 0.05 (-0.01, 0.11)^c^ |
| Executive function | TMT-B (standardised score) | Beavers, 2017 |  |  | 0.00 (-0.06, 0.06)^c^ |
|  | DSST (standardised score) | Beavers, 2017 |  |  | -0.05 (-0.11, 0.01) |
|  | Stroop test (standardised score) | Yu, 2022 |  |  | -0.65 (-1.07, -0.23)^f^ |
|  |  |  |  |  | -0.82 (-1.29, -0.35)^g^ |
|  | Stroop test (standardised score) | Beavers, 2017 |  |  | 0.08 (-0.78, 0.94) ^c^ |

^a^ The results were reported per percentage point increase in HbA1c level unless categories are specified.

^b^ Beta (95% CI) from multiple linear regression models reported unless specified otherwise. Statistically significant values are presented in bold. Higher scores indicate better cognitive function unless specified otherwise.

^c^ Lower scores indicate better cognition.

^d^ Odds ratio (95% CI) from multiple logistic regression models reported.

^e^ *g* calculated through principal component analysis based on TMT-B, BVFT and DSST.

^f^ Results for baseline HbA1c levels

^g^ Results for time-weighted HbA1c levels

Abbreviations: BVFT: Borkowski Verbal Fluency Test, CASI-IRT: Cognitive Abilities Screening Instrument Item Response Theory, DSST: Digit Symbol Substitution Test, MMSE: Mini-Mental State Examination, MoCA: Montreal Cognitive Assessment, RAVLT: Ray Auditory Verbal Learning Test, TMT-A: Trail Making Test part A, TMT-B: Trail Making Test part B

# Supplementary Table 6. Association of HbA1c variability metrics with incident dementia and Alzheimer's disease in people with type 2 diabetes, reporting effect sizes from fully adjusted models.

| **Outcome** | **Variability metric** | **Study** | **Subgroup** | **HR (95% CI)** |
| --- | --- | --- | --- | --- |
| All-cause dementia | SD | Moran, 2024 |  | 1.15 (1.12, 1.17) |
|  | CV | Zheng, 2021 |  | 1.03 (1.01, 1.04)^a^ |
|  |  | Moran, 2024 |  | 2.54 (2.12, 3.05) |
|  | ARV | Moran, 2024 |  | 1.10 (1.09, 1.11) |
| AD | SD | Lee, 2021 | women | 1.14 (1.02, 1.27) |
|  |  |  | men | 1.09 (0.94, 1.26) |
|  | CV | Lee, 2021 | women | 1.01 (1.00, 1.02) |
|  |  |  | men | 1.01 (0.99, 1.02) |
|  |  | Li, 2017 | tertile 1 | Reference |
|  |  |  | tertile 2 | 0.98 (0.82, 1.17)^b^ |
|  |  |  | tertile 3 | 1.32 (1.11, 1.58)^b^ |

Results reported per unit change the variability metric unless specified otherwise.

^a^ Results reported per SD change in CV.

^b^ Results reported compared to tertile 1.

Abbreviations: AD: Alzheimer’s disease, ARV: average real variability, CI: confidence interval, CV: coefficient of variation, HR: hazard ratio, SD: standard deviation

# Supplementary Figure 1. PRISMA flow diagram of identified, screened and included studies

Records identified from:

Embase (n = 4,225)

Medline (n = 2,005)

Reference lists (n = 440)

Records removed before screening:

Duplicate records removed (n = 1,482)

Records marked as ineligible by automation tools (n = 0)

Records removed for other reasons (n = 0)

Records screened

(n = 5,188)

Records excluded

(n = 4,876)

Reports sought for retrieval

(n = 312)

Reports not retrieved

(n = 0)

Reports assessed for eligibility

(n = 312)

Reports excluded:

Wrong patient population (n = 47)

Wrong exposure (n = 45)

Wrong outcome (n = 73)

Wrong study design (n = 71)

Conference abstract (n = 18)

Measures of association not reported (n = 15)

Irrelevant scope (n = 3)

Studies included in review

(n = 40)

Reports of included studies

(n = 40)

**Identification of studies via databases and registers**

**Identification**

**Screening**

**Included**

# Supplementary Figure 2. Galbraith plot showing the individual studies standardised effect size and their precision in relation to the overall effect size

Note: The Galbraith plot displays each study’s standardized effect size against its precision (inverse of the standard error). The meta-analysed effect size and its 95% confidence interval are represented by the slope of the regression line and the boundaries of the shaded area. The slope of the line from each point to the origin reflects the individual study's effect size. Heterogeneity is considered low when 95% of studies fall within the 95% CI of the pooled effect.

# Supplementary Figure 3. Funnel plot visualising published studies for association of hypoglycaemia with dementia

# Supplementary Figure 4. Association of hypoglycaemia with incident all-cause dementia compared to no history of hypoglycaemia in people with type 2 diabetes using effect sizes from fully adjusted models. (a) Meta-analysis of studies evaluating any history of hypoglycaemia. (b) Dose response association of the number of hypoglycaemic events with incident all-cause dementia.

# Supplementary Figure 5. Meta-analysis of association of hypoglycaemia with dementia ranked by calendar years of publications

# Supplementary Figure 6. Cumulative meta-analysis of association of hypoglycaemia with dementia showing the effect adding each study to the pool of meta-analysis.

# Supplementary Figure 7. Assessing the effect of removing one study at a time on the overall estimation of the pooled effect size

# Supplementary Figure 8. Meta-analysis of association of hypoglycaemia with dementia restricted to studies reporting hazard ratios

# Supplementary Figure 9. Meta-analysis of studies assessing the association of hypoglycaemia with incident Alzheimer’s disease in people with type 2 diabetes

# Supplementary Figure 10. Meta-analysis of association of HbA1c as a continuous variable with dementia restricted to studies reporting hazard ratios

# Supplementary Figure 11: Association of HbA1c levels with incident dementia in people with type 2 diabetes using effect sizes from fully adjusted models. (a) Meta-analysis of studies assessing HbA1c as a continuous variable. (b) Summary of studies assessing HbA1c as a categorical variable

# Supplementary Figure 12. Association of HbA1c levels with incident Alzheimer’s disease in people with type 2 diabetes. (a) Meta-analysis of studies assessing HbA1c as a continuous variable. (b) Summary of studies assessing HbA1c as a categorical variable.

Notes: In panel (a), hazard ratios were reported per percentage point increase in HbA1c levels and weights were from random-effects model using empirical Bayes (MP) estimator.

# Supplementary Figure 13. Meta-analysis of association of HbA1c as a continuous variable with Alzheimer's disease restricted to studies reporting hazard ratios

# Supplementary Figure 14. Association of categories of HbA1c with incident vascular dementia in people with type 2 diabetes.

# Supplementary Figure 15. Association of diabetes duration and incident dementia in people with type 2 diabetes using effect sizes from fully adjusted models.

# Supplementary Figure 16. Association of categories of diabetes duration and incident Alzheimer’s disease (AD) and vascular dementia (VaD) in people with type 2 diabetes.
